# Body Composition and Metabolic Syndrome Components on Lipodystrophy Different Subtypes Associated with HIV

**DOI:** 10.1155/2017/8260867

**Published:** 2017-04-30

**Authors:** Livia Bertazzo Sacilotto, Paulo Câmara Marques Pereira, João Paulo Vieira Manechini, Sílvia Justina Papini

**Affiliations:** ^1^Botucatu Medical School, São Paulo State University (UNESP), Botucatu, SP, Brazil; ^2^Ribeirao Preto Medical School, University of São Paulo (USP), Ribeirao Preto, SP, Brazil

## Abstract

HIV-associated lipodystrophy syndrome (HALS) is characterized by body fat redistribution as a consequence of the antiretroviral therapy (ART) introduction, associated with an increased risk of cardiovascular disease development. Subjective diagnosis, classified between three subtypes according to the body region on which fat is lost and/or accumulated, named lipoatrophy, lipohypertrophy, and mixed lipodystrophy, is possibly accompanied with metabolic alterations. Forty people living with HIV/AIDS (PLHA), with clinical diagnosis of HALS and from both genders, were assessed. They performed ambulatorial follow-up and used ART regularly. The main findings were greater lipid profile alterations among women, while no metabolic profile differences were found between the HALS subtypes. The lipohypertrophy group showed major alterations, with higher values for total body fat percent, visceral fat area (VFA), body mass index (BMI), and abdominal and neck circumferences when compared to the other groups. Lean body mass was superior only compared to the mixed lipodystrophy group, and fat mass only compared to the lipoatrophy group. BMI showed strong correlation with the VFA. In conclusion, despite anthropometric alterations related to HALS these individuals present, those are not accompanied with metabolic alterations. Strategies, as behavioral changes and disorders prevention, are important to decrease the risk of cardiovascular disease development.

## 1. Introduction

HIV-associated lipodystrophy syndrome (HALS) is characterized by morphological changes due to the heterogenous disorders to the fatty tissue, leading to body fat redistribution, associated or not with metabolic alterations. Such alterations are consequences of the chronic inflammatory state due to HIV virus infection and therapy usage [1, 2]. HALS is described as a syndrome which considers loss and/or accumulation of fat being subdivided in three types: lipohypertrophy (fat accumulation on the abdominal area and/or located), lipoatrophy (fat reduction in peripherical regions), and mixed lipodystrophy (association of lipoatrophy with lipohypertrophy) [3].

Complications from metabolic and morphological alterations go beyond aesthetic consequences, as they may increase the risk of cardiovascular and pancreatic diseases [4, 5]. It is estimated that the metabolic syndrome (MS) prevalence in people living with HIV/AIDS (PLHA) is high, ranging from 11.2% to 45.4% [6, 7]. The acknowledgement of such alterations must be the main aspect approached in following these individuals, as there is strong evidence that lifestyle changes, including the physical activity incentives and diet quality changes, are fundamental for the prevention and treatment of components involved in MS [8–10].

In individuals who have body fat redistribution, the use of easy access methods for clinical practice which measure body fat is paramount for these individuals to be evaluated quickly and the nutritional diagnosis to be established as soon as possible [11]. Acknowledging the nutritional condition and the early nutritional guidance help in reducing frequency and severity of clinical manifestations. Facing this, the study aimed to verify the association of different HALS subtypes in PLHA with the components of metabolic syndrome and body composition.

## 2. Methods

The sample included PLHA with confirmed clinic diagnosis of any subtype of HALS, which were recruited by convenience (i.e., according to diagnosis confirmation during medical assessment). This was a transversal observational study during which we performed clinical-immunological characterization, anthropometric assessment, nutritional diagnosis, and assessed body composition, metabolic alterations, metabolic syndrome components, and general habits (physical activity, smoking, and alcohol consumption) of PLHA during the follow-up of specialized outpatient service in infectology with a clinical HALS diagnosis from July 2015 to July 2016.

PLHA from both genders took part in the study, in regular ART use for at least a year, ranging from 18 to 60 years old. Pregnant women, people with chronic kidney failure, nephrotic syndrome, B and C virus chronic infection, uncontrolled hypothyroidism, or pace maker, or any other electronic body device users or people unable to perform the body composition assessment were excluded from the study.

The HALS diagnosis and subclassification were subjectively carried out by a physician, which means by detecting characteristic findings during visual screening and physical exam associated with self-report. All individuals had the International Classification Diseases (CID 10) recorded in their electronic medical file.

The clinical-immunological characterization took under consideration the HIV diagnostic period, current ART under use, ART usage period, TCD4+ and TCD8+ cell counting, and viral load. For the anthropometric assessment, weight, height, neck circumference (NC,) and abdominal circumference (AC) measures were used. The nutritional diagnosis was determined according to the body mass index (BMI) values having as reference the figures proposed by the World Health Organization (WHO) for adults [12].

The body composition assessment was performed using the Multifrequency Electrical Bioimpedance Equipment (EB) (Inbody®, S10 model, USA) with straight segmental system. The literature points this method as noninvasive, easy to operate, compact, portable, and inexpensive, suiting to clinical practice due to its efficiency and practicality to assess the total or segmented body fat in HALS patients [13, 14]. The used measurements were lean mass (kg) (LM), fat mass (kg) (FM), total body fat percentage (%) (TBFP), and visceral fat area (cm^2^) (VFA). The preparation for the assessments followed the device manufacturer instruction manual.

The metabolic profile assessment was performed during fasting and considered the plasmatic levels of total cholesterol (TC) and HDL-cholesterol (HDL-c), triglycerides (TGL), and fasting blood glucose (FBG) levels. LDL-cholesterol was calculated using the Friedewald formula (LDL-c = TC − HDL-c − TGL/5) and the non-HDL-cholesterol (nHDL-c) by subtracting TC from HDL-c when it is lower than 400 mg/dL.

For hyperglycemic classification, the FBG value was used, following the proposed levels by the American Diabetes Association [15]. The lipid profile was assessed based on reference values suggested by the American Heart Association, American College of Cardiology, and American Society of Hypertension [16]. For the MS classification, the “Harmonized Syndrome” was used as reference [17], which is defined by the presence of three out of five risk factors, at least.

Initially, a characterization of the population was performed with the descriptive measurements calculation (average and standard deviation) for the anthropometric, bioelectric impedance and biochemical exam data segmenting by gender and lipodystrophy subtype. The comparison between genders was carried out using Student's *t*-test and between lipodystrophy subtypes the analysis of variance followed by Tukey test for multiple comparisons was used.

The categorized variables were assessed for frequency and percentage and the association with gender and subtype were made using* chi*-square test or Fisher exact. Pearson correlations were found between anthropometric and biochemical variables. In all tests, the significance level of 5% or corresponding *p* value was fixed. All analyses were made in the SAS for Windows, v 9.2 program.

The present study was approved by the Ethics Research Committee of the Botucatu Medical School, UNESP (CAAE: 26170414.8.0000.5411), and all participants agreed and signed the informed consent term.

## 3. Results

In the present study, 40 PLHA with HALS were assessed, equally distributed by gender. The average age was 45.5 years ± 8.2 years. The association of two Nucleoside Reverse Transcriptase Inhibitors (NRTI) and two Protease Inhibitors (PI) was major for all groups (47%, 54%, and 70% of the individuals with mixed form, lipoatrophy, and lipohypertrophy, resp.). The average HIV diagnostic period was of 15.3 years ± 6.2 years and the average ART usage period was of 13.8 years ± 5.7 years. The mixed HALS form was the most prevalent one (*n* = 17; 42.5%), followed by lipoatrophy (*n* = 13; 32.5%) and lipohypertrophy (*n* = 10; 25%). From the assessed individuals, 81% (*n* = 34) did not practice any kind of physical activity, 45% (*n* = 18) were smokers, and none of them (100%) referred to alcoholic drink consumption of more than three times a week.

Regarding virologic profile, two individuals had a detectable viral load (values below 60 copies/mL) and the average of TCD4+ and TCD8+ cell counting was 743.7 ± 428 cells/mm^3^ and 968.2 ± 346.7 cells/mm^3^, respectively.

The nutritional diagnosis was performed regarding BMI classification, with an average of 25.6 ± 4.9 Kg/m^2^. Half the individuals were classified as being overweight (*n* = 20; 50%), followed by eutrophic (*n* = 19; 47.5%), and one had a malnourishment status (2.5%). Regarding anthropometric measurements adequacy and body composition, the high percentage of individuals showing alterations in the AC (*n* = 29; 72.5%), TBFP (*n* = 28; 70%), and FM accumulation (55%) measurements is highlighted. However, 60% of the sample (*n* = 24) do not have visceral fat accumulation according to the VFA value. More than half the individuals (*n* = 22; 55%) did not present body LM alterations or NC alteration (*n* = 27; 67.5).

From the total sample, 5% (*n* = 2) had a type 2 diabetes diagnosis and 12.5% (*n* = 5) presented an alteration of glycemic levels. HAS was found in 30% (*n* = 12) of the individuals. Concerning lipid profile, the main findings were increased TGL levels (67.5%; n = 27) and low c-HDL values (57.5%; n = 23). Moreover, lower alteration frequencies were observed in CT, c-LDL, and non-HDL-C levels in 12.5% (n = 5), 10% (n = 4), and 37.5% (n = 15), respectively (Table 1).

The presence of MS was found in 52.5% (*n* = 21) of the assessed individuals, as 47.5% (*n* = 19) did not present MS, ten (25%) had alterations in two components, and eight individuals (20%) had one altered component.

When the sample was divided by gender, a significant difference was observed for all lipid profile values, except HDL-c (Table 2). No statistical differences were observed between genders for the clinical-immunological, anthropometric, and body composition parameters (Tables 3 and 4).

Table 5 presents the distribution of clinical-immunological indicators separated by lipodystrophy subtype. Statistically significant differences were found for TCD4+ cell counting (*p* = 0.0115) between mixed form and lipohypertrophy groups.

In terms of metabolic profile, no statistical difference was found between lipodystrophy subtypes (Table 6).

The main differences found among the HALS subtypes were about the anthropometric measures and body composition (Table 7). There was a statistical difference among the BMI, AC, and NC averages in the mixed form and lipohypertrophy groups (*p* = 0.0007, *p* = 0.0018, and *p* = 0.0062, resp.), as well as between the lipoatrophy and mixed groups (*p* ≤ 0.0001 and *p* = 0.0019, resp.).

Concerning body composition, differences were observed among all the studied parameters. The LM averages differed between the mixed form and lipohypertrophy groups (*p* = 0.0252). The averages of FM and TBFP were different between the lipoatrophy and lipohypertrophy groups (*p* = 0.0008, both). The VFA averages were different between the mixed form and lipohypertrophy groups (*p* = 0.0025) as well as between the lipoatrophy and lipohypertrophy groups (*p* ≤ 0.0001).

Table 8 shows the results of the correlations among anthropometric, body composition, and metabolic indicators. A strong correlation was found between TBCP both with FM and with VFA and between the BMI both with AC and with VFA. BMI showed a moderate correlation with FM as well as with TBCP, similar to the AC to the TBCP. A weak correlation between non-HDL-c with TBCP and the non-HDL-c with AC and VFA was observed.

## 4. Discussion

PLHA follow-up is complex and must rely on a multiprofessional team, as different aspects have alterations in these individuals and are closely related, besides the virus infection itself, to the specific treatment for the disease and life habits. The higher PLHA prevalence with individuals older than 40 years was also found in other studies, with the prevalence of adult individuals being a reflex of the disease specific therapy introduction which does not provide a cure but avoids virus replication and the occurrence of opportunistic diseases, resulting in a higher life expectancy and quality [18–21].

Clinical findings related to the disease aggravation revealed a prevalence of individuals with undetectable viral load and high average T CD4+ lymphocytes count, similar to the ones found in literature [18, 21–24], reflecting the immunological recovery, as well as disease specific treatment compliance. Considering that the present study aimed to assess PLHA alterations in the chronic version of the disease, such results are expected, as they are a reflex of the treatment regularity.

Although ART controls the viral load and promotes the disease control, several studies observe that they are related to important metabolic alterations, such as body composition alteration, glycemic and lipid profile, and MS development risk factors [25, 26]. It is highlighted that the MS development is also related to other factors such as lifestyle, physical activity, and food intake behavior [27]. In the present study, besides physical activity, smoking and alcohol consumption were assessed as they are conditions related to the CVD development. Sedentarism rates found in the study were high, 81%, similar to ones found in another study, which observed a prevalence of 80% [28]. The smoking frequency found in the present study was (43%), lower than an European study [29] (51.5%) but much higher than the estimated prevalence in Brazilian population (8.7%) [30].

The increase in life expectancy as a positive consequence of ART introduction led these individuals to reflect upon the population general characteristics, such as sedentarism which has high rates in general population as well. Considering that such habits are changeable, the results found call our attention for the necessity of physical activity incentives as a risk prevention strategy for the comorbidity development, among DM2, HAS, dyslipidemia, and MS.

The DM2 estimated prevalence in PLHA is low and was observed in the present study (7.1%), being similar to results found in literature [31–33], below the results found for Brazilian adult population (11.9%) [34]. Insulin resistance and DM2 are referred to as adverse effects related to the treatment as well as chronic inflammation related to the HIV infection, made evident by the increase of systemic inflammatory markers.

Recent studies suggest a risk increase for the HAS development among PLHA. In the present study, the 30% prevalence found was similar (31%) to the one found in a transversal study which investigated prevalence and HAS associated factors in PLHA using ART [33]. It is noted that the results found were higher than the ones observed in Brazilian adult population (22.8%) according to data informed by the Risk Factors and Chronic Diseases Vigilance by Phone Inquiry (Vigitel) [35, 36]. These results suggest the necessity of monitoring and preventive measures in this group of individuals as the development of such chronic disease is related to general life habits which are changeable risk factors.

The main lipid profile alterations found in the present study were an increase in TGL and nHDL-C and low serum HDL-C levels. The TGL and HDL-c averages found (198.9 ± 98.8 mg/dL and 44.3 ± 15.3 mg/dL) were similar to another study [27]. However, the prevalence of individuals with hypertriglyceridemia was higher [27]. The use of nHDL-c as an atherogenic profile marker seems to be more efficient than the isolated usage of LDL-c levels in the clinical point of view for MS patients [37]. A study performed to identify the impact of the IP usage compared to the control group also presented a TGL increase and HDL-c levels decrease [38]. The same was observed about MS, with a prevalence of 52.4%, compared to the 21.1% found in a study with PLHA using ART presenting HALS [39].

The prevalence of body alterations with PLHA found in literature is varied as there is no consensus about the definition of HALS adopted by researchers, resulting in different adopted definitions and making it difficult to compare data. Besides, there is the diagnostic limitation as it is performed subjectively, considering the identification of corporal changes by the doctor and/or individual complaints. HALS diagnosis and its subtype classification are important as specific strategies must be considered in each one of them. A study [40] which analyzed a group of articles on the topic found a variation in average prevalence of body alterations between 30% and 62%, and HALS subtypes from 18% to 45% for the lipohypertrophy subtype and from 22% to 38% to lipoatrophy. An Australian study [41] which also assessed HALS in mixed form, lipohypertrophy and atrophy, found a higher mixed form prevalence, followed by lipoatrophy and lipohypertrophy, as well as in the present study though in lower frequency.

Besides HALS diagnostic, the nutritional diagnostic deserves attention in this group of individuals, as the body composition alterations may reflect their nutritional state as observed in this study, in that more than half (52.4%) of the individuals were classified as having an excess of weight (overweight and obesity), with 23.8% obese. These results are similar to the ones found by Soares et al. [42], who, separating individuals by gender, observed weight excess in 45.8% of men and 36.4% women, and lower than the 32.1% found by Mariz et al. [43]. Literature reports that the increase in PLHA obesity prevalence is associated with the significant reduction of opportunistic diseases and HIV chronicity, as ART acts directly halting important stages of HIV replication [44].

In terms of anthropometric assessment, the AC alteration is highlighted, as 52.4% of the assessed individuals presented an increase of such measure, with AC average values higher than another study [45]. Although no statistical difference was found between genders, it is noted that women show a higher value of such measure compared to men. There are literature reports of the relation between estrogen and body fat by means of abdominal lipolysis activity reduction. Women during menopause or climacteric period, when there is a reduction of estrogen, have FM increase and body fat redistribution for the abdominal area, which is a risk factor for the development of cardiovascular events [46, 47].

In the present study, women living with HIV/AIDS showed higher lipid profile alterations compared to men. However, another study [48] which also assessed the lipid profile between genders found statistical differences only for the triglycerides levels. It is worth noting that women have a higher tendency to present higher HDL-c levels compared to men. Larger alterations of lipid profile and FGBL among women living with HIV/AIDS suggest that they may have increased risk for the development of CVD compared to men.

The lack of consensus regarding HALS diagnostic and subclassification criteria makes it difficult to compare all literature data. However, a study which also subclassified the individuals in mixed form, lipohypertrophy, and lipoatrophy also found higher AC, VFA, and TBFP values in the lipohypertrophy group compared to others [41]. In the present study, individuals with the lipohypertrophy type presented higher fat accumulation (BMI, AC, NC, TBFP, and VFA) levels, compared to other HALS types. The FM amount was higher in lipohypertrophy form compared to lipoatrophy, which was expected, as this group characteristic is the fat accumulation in specific regions, whereas lipoatrophy is characterized by the reduction of this body component in specific regions. The lipohypertrophy group presented a higher amount of LM compared to the mixed form group, indicating a preservation of such body component.

Considering that HALS diagnosis is subjective, the usage of precise methods to assess and quantify body composition and identify alterations is important so that they are correctly oriented. No statistical differences were identified among HALS subgroups regarding metabolic profile, only regarding body composition, indicating that the body composition alteration in these individuals is not associated with metabolic alterations.

In the present study, there is a strong correlation of BMI with AC and VFA and moderate correlation of BMI with FM and TBFP and between AC and TBFP as in the study performed by O'Neill et al. [45] which also found a positive association between AC and BMI to the total abdominal fatty tissue, VFA, and abdominal subcutaneous fatty tissue both with men and with women. The identification of such positive associations is important for the usage of the low cost and easy applicability measures and the inclusion in the clinical routine. Such correlations deserve attention as, despite limitations, their usage in clinical practice or in places where more accurate equipment is unavailable for the assessment of such individuals, simple measurements as BMI and AC show a significant correlation with fat accumulation and visceral fat area.

*Study Limitations*. The study limitations include the number of assessed individuals and the comparison to PLAH without HALS and control group which would allow a higher clarity in results.

## 5. Conclusion

In conclusion, the present study showed that although these individuals present alterations in important anthropometric indicators related to the HALS diagnosis, they are not followed by metabolic alterations. New studies including the adipocytokine assessment in this group of individuals must be performed for the better knowledge and clarification of these alterations.

From the obtained results, the necessity of strategy implementation for the PLHA life quality is clear. They must include behavioral changes, identification, prevention, and treatment of chronic diseases. Both body composition and MS diagnostic are fundamental for the PLHA nutritional diagnostic for the early identification of alterations which increase the risk of comorbidity development and cardiovascular diseases.

## Figures and Tables

**Table 1:** Alteration frequency, average values, and minimum and maximum values for glycemic and lipid profile of people living with HIV/AIDS with clinical HIV-associated lipodystrophy syndrome diagnosis.

	Altered		Unaltered		Mean ± SD	Minimum–maximum
	*N*	%	*N*	%		
Fasting blood glucose (mg/dL)	5	12,5	35	87,5	85,2 ± 12,6	(68–130)
Total cholesterol (mg/dL)	5	12,5	35	87,5	192,3 ± 46,5	(125–325)
HDL-cholesterol (mg/dL)	23	57,5	17	42,5	44,3 ± 15,3	(21–88)
Triglycerides (mg/dL)	27	67,5	13	32,5	198,9 ± 98,8	(62–525)
LDL-cholesterol (mg/dL)	4	10,0	36	90,0	108,2 ± 37,8	(54,6–203,2)
Non-HDL-cholesterol (mg/dL)	15	37,5	25	62,5	148,0 ± 44,6	(76–285)

HDL: high density lipoprotein; LDL: low density lipoprotein; mg/dL: milligrams per deciliter.

**Table 2:** Metabolic profile of people living with HIV/AIDS with clinical HIV-associated lipodystrophy syndrome diagnosis separated by gender.

	Female (*n* = 20)	Male (*n* = 20)	*p* value
Fasting blood glucose (mg/dL)	87,7 ± 14,2	82,8 ± 10,5	0,1828
Total cholesterol (mg/dL)	212,7 ± 53,1	171,9 ± 27,1	**0,0037**
HDL-cholesterol (mg/dL)	49,6 ± 14,6	39,0 ± 14,6	0,9970
Triglycerides (mg/dL)	206,8 ± 123,6	190,9 ± 68,1	**0,0128**
LDL-cholesterol (mg/dL)	121,7 ± 43,0	94,7 ± 26,4	**0,0394**
Non-HDL cholesterol (mg/dL)	163,1 ± 50,8	132,9 ± 31,8	**0,0468**

HDL: high density lipoprotein; LDL: low density lipoprotein; mg/dL: milligrams per deciliter.

**Table 3:** Clinical-immunological people living with HIV/AIDS with clinical HIV-associated lipodystrophy syndrome diagnosis separated by gender.

	Female (*n* = 20)	Male (*n* = 20)	*p* value
Age (years)	44,9 ± 7,8	46,2 ± 8,7	0,65
Diagnostic period (years)	15,5 ± 5,8	15,2 ± 6,6	0,59
ART period (years)	14,2 ± 5,3	13,3 ± 6,3	0,69
TCD4+ (cells/mm^3^)	754,7 ± 377,5	732,7 ± 482,8	0,86
TCD8+ (cells/mm^3^)	928,9 ± 346,7	1007,6 ± 351,1	0,49

ART: antiretroviral therapy; TCD+: lymphocyte T; cells/mm^3^: cells per cubic millimeter.

**Table 4:** People living with HIV/AIDS body composition separated by gender.

	Female (*n* = 20)	Male (*n* = 20)	*p* value
Lean mass (Kg)	25,1 ± 5,2	32,7 ± 6,2	0,47
Fat mass (Kg)	25,9 ± 14,5	19,6 ± 10,5	0,18
Total fat percentage (%)	32,7 ± 7,5	23,9 ± 9,3	0,33
Visceral fat area (cm^2^)	97,0 ± 33,6	86,9 ± 38,1	0,59

Kg: kilograms; %: percentage; cm^2^: square centimeter.

**Table 5:** Clinical-immunological people living with HIV/AIDS profile with clinical HIV-associated lipodystrophy syndrome diagnosis separated by HIV-associated lipodystrophy syndrome subtypes.

	Mixed (*n* = 17)	Lipoatrophy (*n* = 13)	Lipohypertrophy (*n* = 10)	*p* value
Age (years)	44,9 ± 8,4	49,3 ± 7,4	41,6 ± 7,6	0,0736
Diagnostic period (years)	15,1 ± 6,0	16,1 ± 7,4	14,8 ± 5,1	0,8681
ART period (years)	13,7 ± 6,0	14,2 ± 5,7	13,3 ± 6,0	0,9556
TCD4+ (cells/mm^3^)	872,2 ± 468,2^ac^	747,3 ± 432,7	520,5 ± 260,0^ac^	0,0586
TCD8+ (cells/mm^3^)	991,4 ± 376,5	934,7 ± 354,4	972,3 ± 314,3	0,9140

ART: antiretroviral therapy; TCD+: lymphocyte T; cells/mm^3^: cells per cubic millimeter.

*Note*. ^ac^Statistic difference between HALS mixed group and HALS lipohypertrophy group.

**Table 6:** Metabolic profile of people living with HIV/AIDS separated by HIV-associated lipodystrophy syndrome subtype.

	Mixed (*n* = 17)	Lipoatrophy (*n* = 13)	Lipohypertrophy (*n* = 10)	*p* value
Fasting blood glucose (mg/dL)	85,7 ± 11,1	81,9 ± 10,4	88,8 ± 17,1	0,3636
Total cholesterol (mg/dL)	195,8 ± 51,6	185,9 ± 48,0	194,5 ± 38,1	0,8403
HDL-cholesterol (mg/dL)	43,1 ± 10,7	47,7 ± 20,1	41,8 ± 15,7	0,6173
Triglycerides (mg/dL)	234,1 ± 131,2	151,7 ± 50,9	200,3 ± 52,1	0,0739
LDL-cholesterol (mg/dL)	105,9 ± 38,0	107,9 ± 39,2	112,6 ± 39,1	0,9082
Non-HDL cholesterol (mg/dL)	152,7 ± 50,0	138,2 ± 44,1	152,7 ± 36,9	0,6410

HDL: high density lipoprotein cholesterol; LDL: low density lipoprotein cholesterol; mg/dL: milligrams per deciliters.

**Table 7:** People living with HIV/AIDS body composition with clinical HIV-associated lipodystrophy syndrome diagnosis separated by HALS subtype.

	Mixed (*n* = 17)	Lipoatrophy (*n* = 13)	Lipohypertrophy (*n* = 10)	*p* value
Lean mass (Kg)	26,7 ± 6,2^ac^	28,2 ± 6,5	33,7 ± 6,5^ac^	**0,0295**
Fat mass (Kg)	23,2 ± 14,9	14,3 ± 5,9^bc^	32,9 ± 7,7^bc^	**0,0012**
Total fat percentage (%)	28,9 ± 8,9^ac^	21,9 ± 8,0^bc^	35,6 ± 6,6^d^	**0,0012**
Visceral fat area (cm^2^)	87,4 ± 34,1^ac^	69,9 ± 20,7^bc^	128,5 ± 26,5^d^	<**0.0001**
Body mass index (Kg/m^2^)	25,0 ± 3,9^ac^	22,4 ± 3,4^bc^	30,9 ± 3,5^d^	<**0.0001**
Abdominal circumference (cm)	90,7 ± 12,7^ac^	84,7 ± 11,2^bc^	107,4 ± 7,9^d^	<**0.0001**
Neck circumference (cm)	35,2 ± 3,4^ac^	34,4 ± 2,2^bc^	39,3 ± 3,6^d^	**0,0015**

Kg: kilograms; %: percentage; cm^2^: square centimeter; Kg/m^2^: kilograms per meter squared; cm: centimeters.

*Note*. ^ac^Statistic difference between HALS mixed group and HALS lipohypertrophy group. ^bc^Statistic difference between HALS lipoatrophy and HALS lipohypertrophy group. ^d^Statistic difference of lipohypertrophy group compared to both mixed form and lipoatrophy groups.

**Table 8:** Pearson simple correlation coefficients among abdominal circumference (AC), visceral fat area (VFA), non-HDL cholesterol (non-HDL-c), fat mass (FM), total body fat percentage (TBFP), and body mass index (BMI).

	AC	VFA	Non-HDL-C	FM	TBFP	BMI
AC	1,00	**0.78**	0.08	**0.46**	**0.58**	**0.86**
VFA		1,00	0.16	**0.72**	**0.89**	**0.83**
Non-HDL-C			1,00	0.24	**0.33**	0.08
FM				1,00	**0.72**	**0.64**
TBFP					1,00	**0.69**
BMI						1,00

*Note*. Highlighted values in bold are *p* < 0.0001. The bold and underlined values are *p* < 0.05.
